# An early onset Gitelman syndrome presenting in a boy with failure to thrive with recurrent hypokalemia and hypomagnesemia: a case report

**DOI:** 10.11604/pamj.2024.49.59.45186

**Published:** 2024-10-29

**Authors:** Ramaning Loni, Noora Almuaili, Hajar Hasan, Naveen Raju, Fareedul Ahmed Hasan, Gabriel Fox, Shatha Hassan Mommad Osman

**Affiliations:** 1 Department of Pediatrics and Pediatric Intensive Care Unit, King Hamad University Hospital, Al Sayh, Bahrain

**Keywords:** Refractory hypomagnesemia, recurrent hypokalemia, metabolic alkalosis, Gitelman syndrome, case report

## Abstract

Gitelman syndrome is an autosomal recessive, chronically salt-losing tubulopathy depicted by renal potassium wasting, hypokalemia, hypocalciuric, hypomagnesemia, metabolic alkalosis, and hyperreninemic hyperaldosteronism with average or low blood pressure. This case report describes a 10-year-old boy who presented with acute respiratory tract infection with respiratory distress, myalgia, generalized muscle weakness, and significant biochemical changes like hypokalemia, hypomagnesemia, and metabolic alkalosis associated with failure to thrive. Further investigations, like genetic testing, showed a SLC12A3 gene mutation, a pathogenic homozygosity variant, proving the diagnosis of Gitelman syndrome. The child needed Intensive Care Unit (ICU) admission for life-threatening electrolyte imbalances with Electrocardiogram (ECG) changes for the acute care and later, requiring a multidisciplinary team approach for the management. The early presentation of Gitelman syndrome in young children must be kept in mind, as it could be missed. The persistent metabolic alkalosis, hypokalemia and hypomagnesemia should raise the concern about the possibility of chronic salt-losing nephropathic conditions.

## Introduction

Gitelman syndrome (GS) is a salt-losing tubule-nephropathy due to a mutated gene encoding sodium chloride and magnesium transporters in the thiazide-sensitive segments of the distal convoluted tubule of the nephron. The predominant features of GS are renal potassium wasting, hypokalemia, metabolic alkalosis, hypocalciuria, hypomagnesemia, and hyperreninemic hyperaldosteronism. GS is also referred to as familial hypokalemia-hypomagnesaemia. Patients may have clinical symptoms of hypokalemia, hypomagnesemia, metabolic alkalosis, and hypocalcemia. In addition, due to long-term electrolyte disorder and acid-base imbalance, GS will lead to growth stunting and renal damage in children [1].

Gitelman syndrome is a benign tubulopathy, usually presented during adolescence or adulthood. The mechanism of GS is due to the mutation of the gene SLC12A3 encoding the sodium chloride co-transporter sensitive to thiazide diuretics localized in the distal convoluted tubules of the kidney. GS is a rare disease worldwide, and the prevalence rate is one to ten for every 40,000 population [1]. The incidence rate in the Asian population is higher than in other populations. Most patients with GS have a good prognosis after effective treatment. GS is often diagnosed by muscle weakness and cramps associated with frequent fatigue or reduced daily activity. Patients with GS do not show symptoms throughout infancy and preschool; their GS is often discovered incidentally [2,3].

However, studies have challenged this idea by emphasizing the disorder's phenotypic variation and potential severity [3]. Although GS is described as an asymptomatic or benign disorder, patients may develop life-threatening complications such as ventricular arrhythmia [4]. Early detection with suitable treatment may prevent potentially life-threatening complications. Therefore, this study aimed to report a case of early clinical presentation of GS like muscle weakness, cramps, failure to gain weight or weight due to refractory hypokalemia, hypomagnesemia with metabolic alkalosis picture.

## Patient and observation

**Patient information:** a 10-year-old boy born of a first-degree consanguineous marriage presented to the hospital with a known case of asthma from five years of age. He had been on regular Inhaled corticosteroid therapy (Budecortisone) and Salbutamol therapy. His younger sibling also has a history of hyper-reactive airway disease. He had multiple chest infections from three years of age with significant hypokalemic episodes requiring ICU care in the past.

**Clinical findings:** the child presented with acute respiratory symptoms and weakness to the hospital. On admission, he was anxious and alert with a normal mentality. He had a noticeable generalized weakness and difficulty in engaging in routine activities. He has severe respiratory distress with a breathing rate of 46 breaths per minute with chest retractions, a temperature of 37.9°C, a heart rate of 130 beats/minute, blood pressure of 98/60 mmHg, and 90% O_2_ saturation in the room air. His anthropometric measurements revealed a height of 122cm (between +2 SD and +3 SD) and a weight of 18.2kg (less than +2 SD), suggesting small for the age. The chest examination showed bilateral wheezes and crackles. A central nervous system examination showed bilateral generalized wasting with a grade of 4/5 in the lower limbs and diminished reflexes. Cardiovascular and abdominal examinations were normal.

**Timeline of the current episode:** the first presentation was in 2017 at the age of three years for acute respiratory infection with hypokalemia. The loss to a follow-up until ten years of age (2024). He has been through four admissions since the beginning of 2024: the first episode in February 2024 for community-acquired pneumonia with severe hypokalemia and metabolic alkalosis. The second admission was in the first week of March 2024 for life-threatening electrolyte imbalances in the form of hypokalemia-induced wide QRS, bradycardia with metabolic alkalosis, and failure to thrive. The third admission was at the end of April 2024 for pneumonia with hypokalemia and metabolic alkalosis. The current admission was in July 2024 for pneumonia with severe hypokalemia, hypomagnesemia, and metabolic alkalosis.

**Figure 1:**
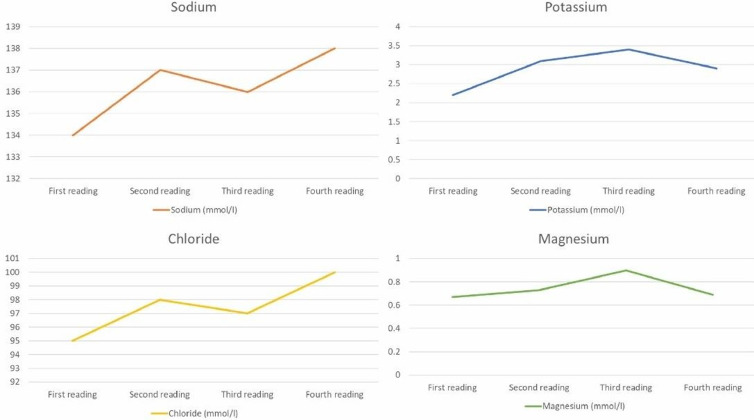
a graphical representation of serum electrolytes with each admission showing persistent hypokalemia and low borderline magnesium levels in the serum

**Diagnostic assessment:** the serial serum electrolytes levels reported persistent hypokalemia (2 to 3.5 mEq/L) (Figure 1) (Table 1), hypomagnesemia (0.6 to 0.8 mEq/L) (Figure 1). Venous blood gas on admission saw a PH of 7.46, PaCO2 of 33.4, HCO3 of 25.5 (Metabolic alkalosis) with a potassium level of 1.69 mEq/L (hypokalemia) (Table 1), and chloride of 95 mEq/L. Further, the urine electrolytes testing showed an increased loss of potassium urine along with hypercalciuria (Table 2). The ultrasonography for the kidneys and urinary bladder was normal. The chest x-ray showed hyperinflated lung fields with perihilar haziness and the respiratory panel was positive for human rhinovirus/enterovirus. The electrocardiogram showed prolonged QT interval with bradycardia due to hypokalemia/hypomagnesemia. After obtaining informed consent, the blood sample, which was sent for the whole exome sequencing test, showed a homozygous variant of uncertain significance in the SLC12A3 gene (Table 3) correlating with the clinical phenotype.

**Table 1:** blood gas analysis

Blood gas	Values
pH	7.50
PaCo2	33.4 mmHg
PaO2	38.5 mmHg
Hco3	25.5 mEq/L
Na+	132.3 mEq/L
K+	1.69 mEq/L

The blood gas analysis shows metabolic alkalosis with hypokalemia

**Table 2:** urinary electrolytes

Sample type	First admission	Fourth admission
Urine Calcium (mmol/l)	0.73 (Normal level 2.5 to 7.50)	0.50 (Normal level 2.5 to 7.50)
Urine Chloride level (mmol/l)	164 (Normal 170 to 250)	Not done
Urine Potassium (mmol/l)	45 (Normal level 0 to 10)	162 (Normal level 0 to 10)
Urine Sodium (mmol/l)	157 (Normal 110 to 165)	160 (Normal 110 to 165)
Random Urine Osmolality (mmol/Kg)	411.7 (Normal 50 to 1200)	584.5 (Normal 50 to 1200)

The table shows hypocalciuria and increased loss of potassium in the urine

**Table 3:** whole exome sequence showing a homozygous mutation for Gitelman syndrome

GENE/REFSEQ	CO-ORDINATE GRCh38	VARIANT (36x)	EXONL/INTRON	VARIANT TYPE	ZYGOSITY/INHERITANCE	OMIM/PHENOTYPE
SLC12A3/NM-0011261082	Chr16;56890297	c.2309G>A p. Gly770Asp	Exon19	Missense	Homozygous/AR	Gitelman Syndrome/OMIM #263800

The table shows a SLC12A3 homozygous mutation suggestive of Gitelman syndrome

**Diagnosis:** severe community-acquired pneumonia with acute asthma exacerbation with severe respiratory distress with failure to thrive with refractory hypokalemia, hypomagnesemia, metabolic alkalosis with hypercalciuria, suggestive of Gitelman syndrome. The differential diagnosis: The loop diuretics and congenital chloride diarrhea were ruled out by history. Cystic fibrosis and Barter syndrome were kept for the differentials here, but further investigations excluded them.

**Therapeutic interventions:** the child needed oxygen via heated humidified high-flow nasal cannula, nebulization therapy with salbutamol and budesonide, regular fluids, potassium, and magnesium corrections. He was started on IV intravenous antibiotics (INJ Ceftriaxone) for 5 days and later stopped after blood cultures were negative. The child was continued on oral potassium and magnesium supplements. After treatment, weakness improved, and serum potassium, magnesium, phosphorus, and pH were stable. The multidisciplinary team involved a pediatric pulmonologist, dietician, pediatric nephrologist, geneticist, and child psychologist.

**Follow-up and outcome of interventions:** regular follow-up was recommended for the family. After discharge, the patient was seen in the pediatric outpatient clinic, and the medication dosage was adjusted to keep blood potassium and magnesium within the normal range (serum K+ 3.5 mmol/L and serum Mg+ 0.7 mmol/L). The inhaled corticosteroids were continued for asthma and recommended following up with a pediatric pulmonology clinic. His failure to thrive was managed by a team that included a dietician and a child psychologist. One month after discharge, the child's weight increased by 1kg, and the patient's psychological and emotional state remained stable. While he was taking the medication, no other complications or side effects of the drugs were noticed. Therefore, we concluded that the treatment's effect and the patient's prognosis were good. At the three-month follow-up, he had gained 2kg of weight and 2cm in height. He is scheduled for routine follow-up and monitoring of his electrolyte, respiratory, and growth status with the pediatric pulmonology and nephrology outpatient departments.

**Patient perspective:** both parents and the child felt better psychologically and medically. The child is hoping to now attend school regularly.

**Informed consent:** the parents signed the consent form after explaining the need for publication (e.g. to improve awareness and knowledge among the medical fraternity).

## Discussion

The child's presentation started at 3 years of age with hypokalemia; this is one of the earliest documented presentations of Gitelman syndrome. However, the diagnosis was missed because the child did not attend a follow-up appointment. Later, at the age of 10 years, he presented with an acute respiratory infection, asthma exacerbation, easy fatigability and muscle weakness (mainly including muscle aches and pains intermittently) and had failed to grow, indicated by his short stature. His growth parameters, like weight (18.2kg) and height (122cm), were below the third centile as plotted on the proper growth chart according to his age. He has had multiple episodes of asthma exacerbation that needed hospital admission for oxygen therapy and other supportive management. Most GS patients present with mild, non-specific symptoms in adolescence or adulthood [1]. In our study, the earliest we noticed was hypokalemia at three years of age, as compared to other case reports by Chen *et al*. [2], where he discovered it as early as three weeks of age in preterm twin babies and two years of age, respectively.

Moreover, this child had a history of palpitations, for which an ECG showed prolonged QT, worsened by severe hypokalemia and hypomagnesemia. Worth mentioning here is that this patient had had multiple earlier emergency visits and four earlier hospital admissions. His first emergency visit was at the age of three years when he presented with a history of fever and flu-like symptoms. At that time, routine basic laboratory investigations showed low serum electrolyte values, including sodium of 133 mEq/L, potassium of 2.12 mEq/L, chloride of 93 mEq/L, and carbon dioxide of 26.71 mm Hg. His laboratory findings still showed persistent low potassium value (Figure 1), (Table 1). These clinical manifestations and laboratory findings are comparable to other studies [2,3]. The pathogenesis of GS is due to an SLC12A3 gene mutation encoding the sodium chloride co-transporter sensitive to thiazide diuretics localized in the distal convoluted tubules of the kidney, resulting in the malabsorption of Na^+^and Cl^-^in the distal convoluted tubules [2-4]. The whole exon sequencing report of the child (Table 3) revealed a SLC12A3 gene mutation, already a proven pathogenesis pathway for the Gitelman syndrome's presentation, as given by Riveira-Munoz *et al*. [5]. Therefore, the main clinical manifestations of Gitelman syndrome patients are hypokalemia, hypomagnesemia, hypocalcemia, metabolic alkalosis, asthenia, tetany, and growth retardation in children. A few GS patients also have proteinuria and renal function damage [2,5].

Hypokalemia is a common laboratory finding, especially in hospitalized patients. Therefore, laboratory testing should be obtained to decide the etiology, such as decreased intake, increased intracellular uptake, gastrointestinal loss, or increased urinary loss. Congenital chloride diarrhea and chronic diuretic therapy were ruled out by history, and minor cases of cases could be due to primary hyperaldosteronism and thyrotoxicosis [6-8]. Due to this reason, further tests are needed, such as blood and urine tests, including venous blood gas, for serum levels of sodium, chloride, magnesium, aldosterone, renin, calcium, and acid-base status. The serum magnesium level will be low in Gitelman syndrome, which is expected in Barter syndrome. The urine calcium concentration decreases in Gitelman syndrome as compared to high urinary calcium level in Barter syndrome, as shown in Table 2. The child also has increased potassium loss in the urine (Table 2). Genetic disorders of the tubules are a source of hypokalemia caused by increased urinary potassium loss. Because of genetic mutations in the genes encoding tubular transport proteins involved in sodium reabsorption, patients' sodium absorption is disrupted, leading to increased distal delivery of sodium, which results in metabolic alkalosis and hypokalemia. The average age at onset and diagnosis of Gitelman syndrome is 20.0 ± 8.4 years and 23.6 ± 10.4 years, respectively [9].

The first diagnosis of Gitelman syndrome has primarily been based on clinical presentations and the biochemical analysis of blood and urine, although other differentials like Barter syndrome and cystic fibrosis were considered. The treatment of Gitelman syndrome aims to improve the patient's symptoms and quality of life. The primary treatment method is lifelong electrolyte replacement therapy. In addition to dietary supplements in daily life, potassium and magnesium supplements are used to support the stability of serum potassium and magnesium [1-9]. The typical presentation of GS could be missed sometimes during early childhood which differentiates this case report from other studies.

## Conclusion

Gitelman syndrome is a rare disease that can easily be misdiagnosed or missed due to a lack of awareness and access to clinical pictures. When patients present with hypokalemia, hypomagnesemia, and metabolic alkalosis that are difficult to correct, the possibility of Gitelman syndrome should be considered. Clinical correlation with laboratory reports is necessary. Gene sequencing is needed to confirm the disease and start the electrolyte replacement therapy to improve the patient's clinical symptoms and quality of life.
